# Characteristics of Patients Infected with Norovirus GII.4 Sydney 2012, Hong Kong, China

**DOI:** 10.3201/eid2004.131457

**Published:** 2014-04

**Authors:** Martin C.W. Chan, Ting F. Leung, Angela K. Kwok, Nelson Lee, Paul K.S. Chan

**Affiliations:** The Chinese University of Hong Kong, Hong Kong, China

**Keywords:** hospitalization, viruses, norovirus, infant, norovirus, GII.4 Sydney 2012, viral burden, viral load, Hong Kong, China

## Abstract

Norovirus GII.4 Sydney 2012 has spread globally since late 2012. We report hospitalization of patients infected with this strain skewed toward infants and young children among 174 cases during August 2012–July 2013 in Hong Kong, China. This group had higher fecal viral load (≈10-fold) than did older children and adults.

Norovirus infection is a leading cause of acute gastroenteritis in all age groups in industrialized and developing regions (*1**–**3*). In late 2012, a new norovirus strain of genogroup II, genotype 4 originating in Sydney, Australia (GII.4 Sydney 2012), became the predominant norovirus strain and caused a severe norovirus season globally (*4**–**6*). In Hong Kong, China, this strain caused an off-season communitywide surge in acute gastroenteritis during summer (July–October) 2012 (*6**,**7*). We report hospital admission of persons with this novel strain, which was skewed toward infants and young children for whom fecal viral load of this strain were higher than for patients in other age groups.

## The Study

This 1-year prospective study was conducted during August 2012–July 2013. The study site, Prince of Wales Hospital, is a 1,400-bed acute care and general teaching hospital that serves a population of ≈600,000 (9% of the Hong Kong population). Fecal specimens were routinely collected on the day of admission from patients who had acute gastroenteritis and were suspected of having norovirus infections, but laboratory testing for norovirus was performed in weekly batches. Patients whose test results were positive on Monday of each week were enrolled in this study. We measured concentration of viral RNA in fecal specimens using quantitative reverse transcription PCR (qRT-PCR) (SuperScript III One-Step RT-PCR System with Platinum Taq, Life Technologies, Grand Island, NY, USA) and using primers and TaqMan probe targeting open reading frame 1/2 junction as described (*8*). Cycle threshold (C_t_) was used as a proxy measure of fecal viral load. A positive control for which C_t_ value was known was included in each test run to check for batch-to-batch variation. We performed genotyping by generating an ≈500-bp amplicon that contained a partial RNA-dependent RNA polymerase and a partial major capsid gene. We used G1FF/G1SKR and G2FB/G2SKR primers as appropriate (*8*), then performed sequencing and phylogenetic analysis using the norovirus genotyping tool (www.rivm.nl/mpf/norovirus/typingtool). We extracted statistics on the catchment population of the hospital from the Hong Kong 2011 Population Census (www.census2011.gov.hk/en/district-profiles.html). We used the nonparametric Mann-Whitney U test for univariate comparison of continuous variables and Fisher exact test for categorical variables. We performed statistical analyses using Prism 5.04 (GraphPad). Two-tailed p values <0.05 were considered statistically significant. Ethics approval was obtained from the institutional clinical research ethics committee (reference number CRE-2013.330).

We analyzed specimens of 174 patients admitted with laboratory-confirmed norovirus infection (Table). The number of cases peaked in September 2012, then declined to a low level for the remaining study period. Of the 174 norovirus isolates collected, genotyping was successful in 140 (80.5%). Failure in genotyping was caused by low viral load; no new strains were identified. GII.4 Sydney 2012 strain accounted for most (125 [89.3%]) of the typed cases, followed by GII.3 (4 [2.9%]), and GII.6 (3 [2.1%]) (Table). The previous predominant strain, GII.4 New Orleans 2009, was not detected, and the GII.4 2006b strain was identified in 2 cases. Half of the GII.4 Sydney 2012 cases were selected for RNA-dependent RNA polymerase sequencing; no evidence of a recombinant GII.4 Sydney 2012 strain, which was reported from Denmark and Italy, was observed (*9**,**10*). Non-GII.4 strains were observed more frequently after the epidemic (November 2012–July 2013) than during the epidemic (August–October 2012) (23.5% vs 1.1%; p<0.0001).

**Table:** Monthly distribution of norovirus cases, Hong Kong, China, August 2012–July 2013*

Year, month	All	No. typed	GII.4 Sydney 2012, no. (%)	Other GII.4 strains, no. (strain)	Other genotypes, no. (type)
2012					
Aug	33	32	32 (100)	0	0
Sept	46	40	39 (98)	1 (2006b)	0
Oct	26	17	15 (88)	1 (2006b)	1 (GII.6)
Nov	11	9	9 (100)	0	0
Dec	6	2	2 (100)	0	0
2013					
Jan	12	9	6 (67)	0	1 (GII.6), 1 (GII.8), 1 (GII.13)
Feb	7	6	5 (83)	0	1 (GI.8)
Mar	5	3	3 (100)	0	0
Apr	11	10	4 (40)	0	1 (GI.4), 1 (GII.6), 3 (GII.3), 1 (GII.13)
May	6	4	3 (75)	0	1 (GII.3)
Jun	5	4	3 (75)	0	1 (GII.13 and GII.17 coinfection)
Jul	6	4	4 (100)	0	0

*GII, genogroup II; subsequent number is genotype.

**Figure 1:**
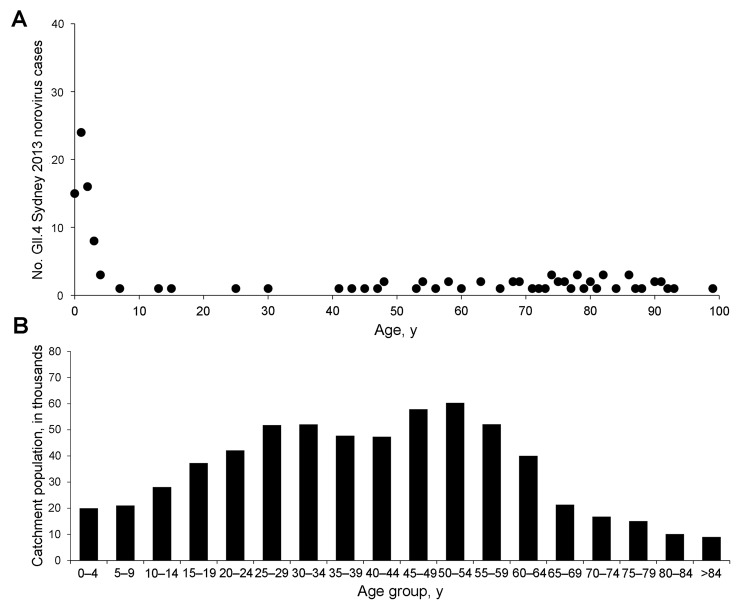
Age distribution of patients with A) norovirus strain of genogroup II, genotype 4 (GII.4 Sydney 2012) and B) study catchment population for the Prince of Wales Hospital area, Hong Kong, China, August 2012–July 2013.

**Figure 2:**
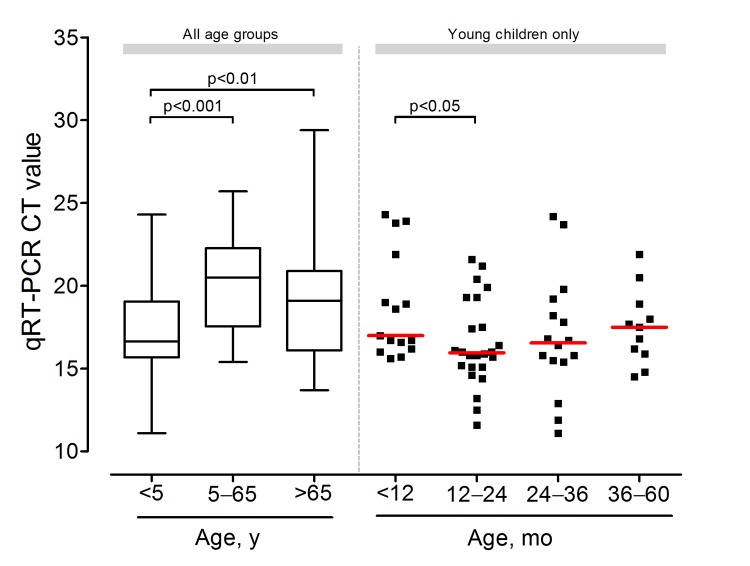
Box-plot of fecal viral load of patients with norovirus strain of genogroup II, genotype 4 (GII.4 Sydney 2012) at hospital admission, Hong Kong, China, August 2012–July 2013. Horizontal lines indicate median values. C_t_, cycle threshold; GII.4, genogroup II genotype 4; qRT-PCR, quantitative reverse transcription PCR.

The age distribution of persons with GII.4 Sydney 2012 and the catchment population of this study are shown in Figure1, panels A and B, respectively. The ages of study patients with GII.4 Sydney 2012 spanned all age groups from infants to persons >90 years of age. The median age was 3 years (interquartile range [IQR] 1–74 years). Median age of patients with GII.4 Sydney 2012 and all study patients did not differ significantly (p = 0.96). The D’Agostino-Pearson normality test indicated that the age distribution did not follow a Gaussian distribution (p<0.0001) but showed a strong positive skew toward infants and children <5 years of age. Among the 125 GII.4 Sydney 2012 cases, 66 (52.8%) were infants or children <5 years of age; the next largest group comprised adults >65 years of age (39 [31.2%]). A similar age distribution was observed when all study cases were analyzed. The GII.4 Sydney 2012 fecal viral load on initial examination is shown in Figure 2. Higher viral load was observed among infants and children <5 years of age and adults >65 years of age. The median viral load, as reflected by C_t_, was highest for infants and children <5 years, next highest for adults >65 years of age, and lowest for the remaining group, respectively (16.7 [IQR 15.7–19.1] vs 19.1 [16.1–20.9] vs 20.5 [17.6–22.3]). The median viral load of infants and children <5 years was 5.5-fold (p<0.001) and 14.4-fold (p<0.01) higher than that of adults >65 years of age and the remaining age group, respectively. Among infants and young children, the highest median viral load was observed in those 12–24 months of age, which was the group with the highest number of admissions (Figures 1 and 2). When all study cases were included, the viral load distribution was similar.

## Conclusions

Noroviruses cause acute gastroenteritis in all age groups. However, most epidemiologic studies have focused either on community outbreaks or on a specific age group such as children; a recent meta-analysis concluded that studies involving all age groups in hospitals are limited and few (*3*). Our study design element of selecting no specific age group enabled us to determine that infants and young children represented approximately half of the hospitalized case-patients with norovirus gastroenteritis during the 1-year study period, during which the newly emerged GII.4 Sydney 2012 strain predominated. Our findings agree with evidence that infants and young children are likely to have the highest rate of infections in health care and community settings (*11**–**13*). In our study, infants and young children had the highest fecal viral load, compared with other age groups. Similarly lower C_t_ in children was reported in a historical cohort of norovirus gastroenteritis in the United Kingdom (1993–1996) (*14*). The higher viral load may relate to delayed viral clearance related to immune naivety. Higher fecal viral shedding also supports a recent mathematical model suggesting that children aged <5 years are more infectious than older children and adults (*15*).

Our study has limitations. Because only patients with diagnoses of norovirus made on a specific weekday each week were enrolled, a crude number of hospitalized patients was used instead of population-based incidence to estimate disease incidence. This limitation does not jeopardize our conclusions because our catchment population pyramid is constrictive, showing fewer younger persons. Second, because information was not complete about specimen collection times after each patient's illness onset, we could not adjust viral load data for specimen collection date; therefore, we cannot rule out a possible bias that might have been introduced if younger children were brought to the hospital earlier during their illness than older patients. Nevertheless, our finding that the most prevalent age group of hospitalized norovirus-infected patients showed the highest viral load implies that plans should be made and implemented for nosocomial infection control of this norovirus strain. Finally, whether our findings apply to other norovirus strains remains unanswered.

We showed that infants and children aged <5 years represent most patients hospitalized for norovirus (GII.4 Sydney 2012) gastroenteritis, and they might have higher viral load than infected persons in other age groups. Our findings may provide public health insights into understanding norovirus transmission in the community.
